# Low-Cost Spinning Disk Confocal Microscopy with a 25-Megapixel Camera

**DOI:** 10.3390/s25237183

**Published:** 2025-11-25

**Authors:** Guy M. Hagen, Brian Lewis, Summer Levis, Joseph R. Hamilton, Tristan C. Paul

**Affiliations:** UCCS BioFrontiers Center, University of Colorado Colorado Springs, 1420 Austin Bluffs Parkway, Colorado Springs, CO 80918, USA

**Keywords:** confocal microscopy, spinning disk, fluorescence, CMOS camera

## Abstract

Spinning disk confocal microscopy enables fast optical sectioning with low phototoxicity but is often inaccessible due to high hardware costs. We present a lower-cost solution using a 25-megapixel machine vision CMOS camera and a custom-built spinning disk. This camera uses a back-illuminated sensor with high quantum efficiency and low read noise. High-resolution images of Thy1-GFP mouse brain slices, *Drosophila* embryos and larvae, and H&E-stained rat testis verified performance across 3D tissue volumes. The measured resolution was 215.8 nm in X, Y and 521.9 nm in Z with a 60×/1.42 NA objective. The custom disk, made with 18 µm pinholes (180 µm pitch) on a chrome photomask and mounted to an optical chopper motor, enables stable, near-telecentric imaging at lower magnifications. Micromanager software integration allows synchronized control of all hardware, which demonstrates that affordable CMOS sensors can potentially replace sCMOS in spinning disk microscopy, offering an open-access, scalable solution for advanced imaging.

## 1. Introduction

Fluorescence microscopy is one of the most important laboratory methods in use today in many areas of biological and biomedical research. Research-grade fluorescence microscopes and their related light sources and detectors are prohibitively expensive in some situations, so finding lower-cost alternatives is an important goal. Innovative approaches include open-access hardware designs for brightfield microscopy [1], for widefield fluorescence microscopy [2,3], for light-sheet microscopy [4], and for two-photon microscopy [5]. Lower-cost approaches for super-resolution microscopy include those for structured illumination [6] such as openSIM [7], and Open-3DSIM [8], or for single molecule localization such as miEye [9]. Open-source software like Micromanager 2.0 [10] for microscope control and data acquisition allows researchers to assemble a complete imaging system. Open-source software for data analysis is also vital, with ImageJ [11] and FIJI [12] being the most commonly used. A variety of plugins for the ImageJ environment allow specialized analysis algorithms to be employed [13,14] and allow for data compatibility between systems [15].

Spinning disk confocal microscopy [16,17] is a camera-based method in optical sectioning fluorescence microscopy which is often used in live cell imaging studies [18,19,20]. Spinning disk systems have been introduced by Conchello and Lichtman [21], by Kino [22], by Wilson [23,24], and by others. Advanced multiphoton spinning disk systems have also been introduced [25]. Super-resolution imaging is possible using image scanning microscopy methods [26]. Commercially, the microlens-enhanced design produced by Yokogawa is the most commonly used and is the most commercially successful [27]. Unfortunately the Yokogawa device can be very expensive when considering a complete system, especially for the newest designs [28].

For this work, we followed a recent design for a low-cost, home-built spinning disk confocal microscope [29], with a few modifications as described below. Our system includes a custom-made glass disk with a pinhole array and lower-cost lasers. The scientific CMOS cameras typically used in spinning disk confocal microscopy have high quantum efficiency and low noise together with fast readout, but are expensive. For detection we therefore used a CMOS camera intended for machine vision purposes. Previous machine vision cameras in this category were less suitable for low-light imaging conditions such as those present in fluorescence microscopy, but newer CMOS cameras have improved quantum efficiency and reduced noise, which offer large improvements in sensitivity and usefulness for this application. Taken together, our system offers an alternative to commercial spinning disk confocal microscopy designs with much lower cost and, because of the open architecture, improved flexibility for a variety of imaging research.

## 2. Materials and Methods

### 2.1. Samples

We imaged an optically cleared coronal mouse brain slice in which a subset of neurons express green fluorescent protein (GFP). The slice was approximately 200 μm thick and was obtained from SunJin Lab (Hsinchu City, Taiwan). This supplier used a *Thy1*-GFP mouse strain [30], which the supplier prepared as follows:

1. cardiac perfusion with cold, freshly prepared 4% paraformaldehyde (PFA),
2. fixation of the dissected brain with a 4% PFA solution on an orbital shaker overnight at 4 °C followed by washing three times with phosphate-buffered saline (PBS) at room temperature,
3. sectioning the brain manually using a vibratome followed by clearing of the slice with RapiClear 1.52 (SunJin Lab) overnight at room temperature,
4. mounting of the cleared sample with fresh RapiClear 1.52 reagent in a 0.25 mm-deep iSpacer microchamber (SunJin Lab).

A second sample was a *Drosophila melanogaster* embryo expressing GFP- labeled histones [31]. The fly stock (#32045) was acquired from Bloomington drosophila stock center. A third sample was a *Drosophila melanogaster* larvae expressing GFP-labeled dendritic arborization (da) sensory neurons [32,33]. This fly line was a kind gift from Dr. Eugenia Olesnicky (University of Colorado, Colorado Springs Biology Department, Colorado Springs, CO, USA). *Drosophila* lines were maintained under standard conditions.

We also imaged commercially available prepared slides containing rat testis tissues that were stained with hematoxylin and eosin (H&E, slide number 31-6464, Carolina Biological, Burlington, NC, USA). This preparation is highly fluorescent and contains tissues with noticeable 3D structures.

### 2.2. Microscope Setup

The system is based on an Olympus (Olympus, Tokyo, Japan) IX83 motorized inverted microscope. We used Olympus objective lenses UPLSAPO 20×/0.85 NA oil immersion, WD 0.17 mm, and UPLXAPO 60×/1.42 NA oil immersion, WD 0.15 mm. Sample XY movements were controlled by a motorized stage (Applied Scientific Instrumentation, Eugene, OR, USA) while focusing was controlled by the IX83 microscope focus motor.

We used 445 nm (for GFP excitation) and 532 nm (For H&E excitation) lasers which were combined with dichroic mirrors and coupled into a multimode fiber (1 mm diameter, Thor Labs, Newton, NJ, USA, part number M35L02). To reduce speckle patterns from the multimode fiber, we shook the fiber with a home-made shaker based on a 120 mm computer fan. We imaged the end of the fiber on to the spinning disk with a microscope objective, (Olympus 40× UPL) together with a 175 mm achromat lens (Edmund Optics, Barrington, NJ, USA). This results in an evenly illuminated field, except for the minimal remaining laser speckle [34]. The laser was reflected to the sample with a dual-band dichroic mirror (part ZT442/532rpc, Chroma, Bellows Falls, VT, USA).

The pinhole array present on the spinning disk breaks the expanded laser beam up into a set of beamlets (approximately 4500 per camera field of view). These beamlets are focused onto the sample where they excite fluorescence. The size of each beam at the sample plane would be the size of the pinholes (18 µm in our case, described in Section 2.3 below) divided by the objective magnification. At 20× magnification the beam diameter at the sample plane would be expected to be 18 µm/20 = 0.9 µm. At 60× magnification the expected beam diameter at the sample plane would be 18 µm/60 = 0.3 µm.

**Figure 1:**
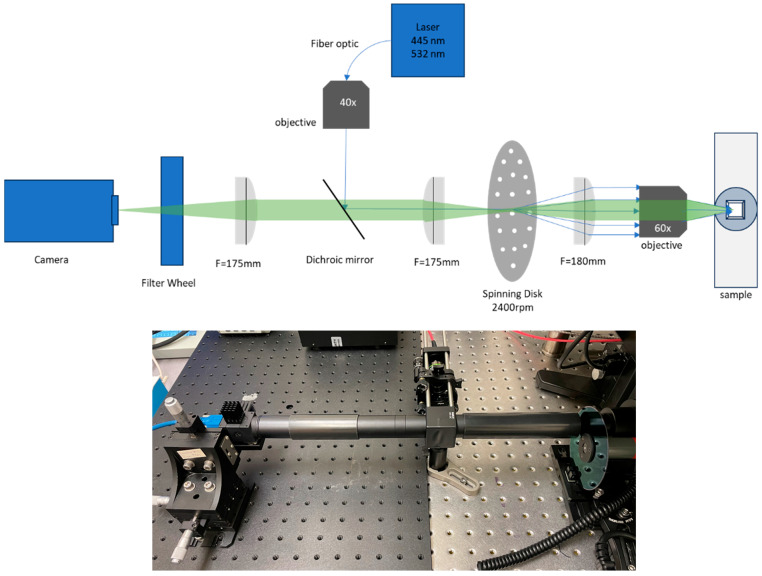
(**top**) Setup for spinning disk confocal microscopy. A 40× objective and 175 mm lens image the end of a multimode fiber optic onto the spinning disk which is positioned in the primary image plane of the microscope. (**bottom**) Photograph of the system during practical use with filter wheel removed.

Fluorescence from the sample was imaged onto the spinning disk with the Olympus microscope and internal tube lens, then relayed onto a CMOS camera (Sony IMX540 sensor within the Blackfly S camera, Teledyne FLIR, Thousand Oaks, CA, USA) with a pair of 175 mm focal length achromatic lenses (Edmund optics). A filter wheel (Lambda 10B, Sutter Instruments Novato, CA, USA) housed the emission filters (ET500/50 for GFP and ET575/50 for H&E staining, Chroma). We used Micromanager software [10] to acquire the images with the 25 MP camera. Micromanager controls the camera, Olympus IX83 *Z*-axis drive, and filter wheel. Our setup is shown in Figure 1. In some experiments we used a Zyla 4.2+ camera (Andor, Belfast, UK) to compare its performance with the IMX540 sensor.

### 2.3. Spinning Disk Design

Front range photomask (also known as Arizona Micro, Las Vegas, NV, USA) produced the chrome-coated borosilicate glass disk based on a CAD file we provided. A Mathematica (Wolfram Research, Champaign, IL, USA) program provided by Halpern et al. [29] was used to produce the disk design. The photomask consists of a reflective chrome coating with an array of pinhole openings arranged in Archimedean spirals. Front range photomask also applied a broad-band anti-reflection coating. The glass disk is mounted on a Stanford Research Systems (Sunnyvale, CA, USA) model SR540 optical chopper. This was chosen because optical choppers are designed for constant rotation speed. The speed is constantly monitored and adjusted by the chopper using the timing notches. In our case, the timing notches were openings in the chrome photomask along the outer edge of the disk.

The disk design is based on the work of Halpern et al. [29] with a few changes. Halpern et al. uses a disassembled computer hard disk drive as the motor, here we used the optical chopper. Halpern et al. has a disk design in which there are multiple sectors radially, so that different pinhole diameters and spacings can be accommodated on the same disk. We changed this to have only a single pinhole diameter and spacing on the disk. This means that we can use (nearly) 1:1 imaging throughout whereas Halpern et al. uses some extra magnification because each sector is smaller than the field of view of the camera chip. This means our optical setup is a bit longer (because of the focal lengths of lenses), but we have a nearly telecentric (no change in magnification when changing focus) design.

The optimal pinhole diameter in a spinning disk confocal microscope is given by [29] d=1.22λ∗M/NA, where λ is the wavelength of light, *M* is the magnification and *NA* is the numerical aperture. Table 1 shows the optimum pinhole size for a range of (Olympus) objective magnifications and NA for a wavelength of 515 nm, e.g., for imaging GFP.

**Figure 2:**
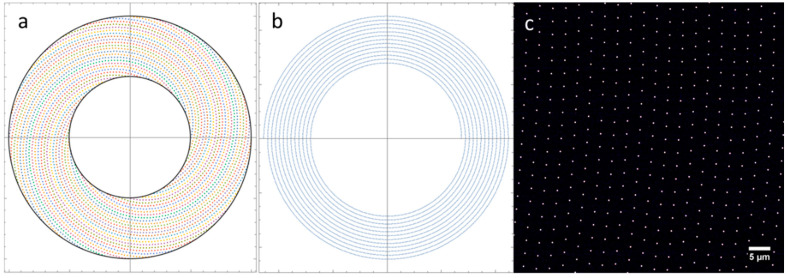
Schematic of spinning disk design. (**a**) Set of spirals, (**b**) single spiral, and (**c**) image from the camera when the disk is stopped showing pattern of pinholes in the middle of the sector as imaged by a 60×/1.42 oil immersion objective.

Often, an inter-pinhole spacing of 10× the pinhole diameter is chosen [29]. In the final design, we chose a pinhole diameter of 18 µm and spacing of 180 µm. This was nearly optimal for a 30×/1.05 NA objective but is a good compromise for use between 20× and 60× magnifications. Halpern et al. [29] designed their disk to be optimal for 100× or 60× objectives. Figure 2 shows a schematic of the disk design including the set of spirals (Figure 2a), a single spiral (Figure 2b), and an image from the camera when the disk is stopped (Figure 2c). The sample in this case was a thin, uniform fluorescent film containing rhodamine dye.

**Figure 3:**
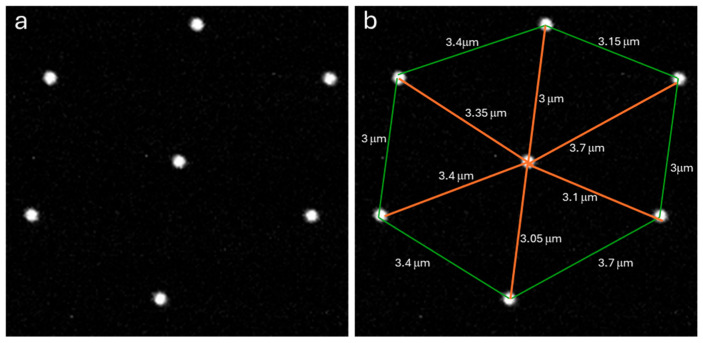
Enlarged view of the center of Figure 2c showing the pinhole pattern. (**a**) Image of the pinhole pattern. (**b**) Measurements of the inter-pinhole distances using a 60×/1.42 oil immersion objective.

Figure 3a shows group of pinholes from the center of the image in Figure 2c. This “unit cell” was measured in Figure 3b to determine the spacing between the pinholes at the sample plane in an actual camera image.

### 2.4. Machine Vision Camera

The sensor we used was the Sony IMX540 as implemented in the Blackfly S camera from Teledyne FLIR. This sensor is part of the Sony Pregius S series of back-illuminated global shutter CMOS sensors. The IMX540 has previously been used in stereo vision applications [35]. With their low-cost CMOS cameras like the one used here are useful in multi-camera setups such as the multi-plane structured illumination microscope used by our group [36]. Table 2 summarizes the parameters of the machine vision camera, and a camera more commonly used in spinning disk confocal microscopy, the Andor Zyla 4.2+ scientific CMOS.

This Sony IMX540 sensor has smaller pixels than are typically used in microscopy. This has the disadvantage that each pixel is less sensitive by the ratio of the pixel areas between the two cameras (6.5 µm)^2^ vs. (2.74 µm)^2^, a factor of 5.63. However, the advantage of a smaller pixel size is that the image will be oversampled at lower magnifications.

The resolution of a widefield fluorescence microscope is given by *d* = 0.61 *λ/NA*, where *d* is the distance between two objects that can just be resolved according to the Rayleigh criterion. To achieve proper sampling, we need at least two pixels covering the point spread function. That is, the expected resolution divided by the back-projected pixel size should be at least 2.0. Table 3 shows, for a variety of (Olympus) objectives, the expected resolution for λ = 515 nm, and the back-projected pixel size for the two different cameras (Sony IMX540 and Andor Zyla). The back-projected pixel size refers to the size of a camera pixel projected to the sample through a particular objective. For example, the Sony IMX540 sensor has a pixel size of 2.74 µm. When using at 10× objective, the back-projected pixel size would be 0.274 µm (274 nm). Table 3 also shows the sampling rate, calculated as the expected resolution divided by the back-projected pixel size. We can see that, in the widefield case, using the Sony IMX540 sensor allows oversampled imaging even at 10× magnification. Here we are using spinning disk confocal microscopy, which (when not using methods such as optical photon reassignment) does not typically achieve lateral resolution better than a widefield microscope [28].

### 2.5. Low-Cost Lasers

We used two lasers, a 2 W, 532 nm DPSS laser (Dragon laser, Changchun, China), and a 1.5 W, 447 nm laser (a low-cost (approx. USD45.00) diode laser acquired through eBay). The lasers can be rapidly toggled on and off with 0–5 V signals, useful for blanking the lasers in between camera exposures. We have found these lasers to be reliable and to have sufficient stability for our purpose. The higher powers are useful in this setup because we do not utilize the microlens-enhanced design as is found in commercial spinning disk units from Yokogawa [28]. The microlens-based design is a much more difficult construction because the microlenses must be positioned accurately above the pinholes and because a dichroic mirror must be positioned in between the two disks.

## 3. Results and Discussion

### 3.1. Characterization of the Camera

**Figure 4:**
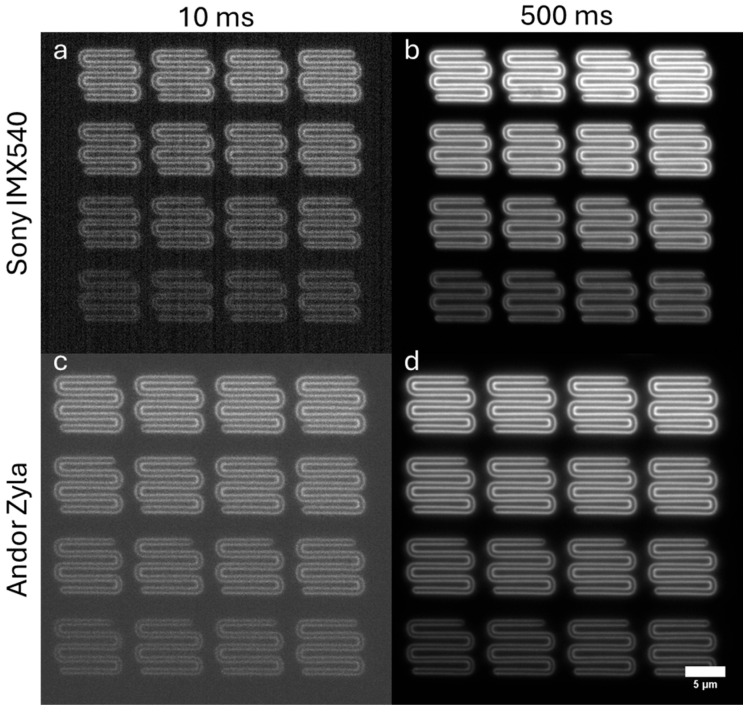
(**a**) 25 MP CMOS camera, 10 ms exposure, (**b**) 25 MP CMOS camera, 500 ms exposure. (**c**) Andor Zyla camera, 10 ms exposure, (**d**) Andor Zyla camera, 500 ms exposure. Objective: Olympus 100X/1.40 NA UPLSAPO oil immersion.

To characterize the two cameras, we imaged an intensity gradient pattern on an Argo-SIM slide (Argolight, Pessac, France). The intensity gradient pattern consists of 16 squares having different fluorescence intensity levels following a linear evolution. This is shown in Figure 4. The Andor Zyla camera has noticeably lower noise levels at 10 ms exposure. At 500 ms the images are more comparable.

**Figure 5:**
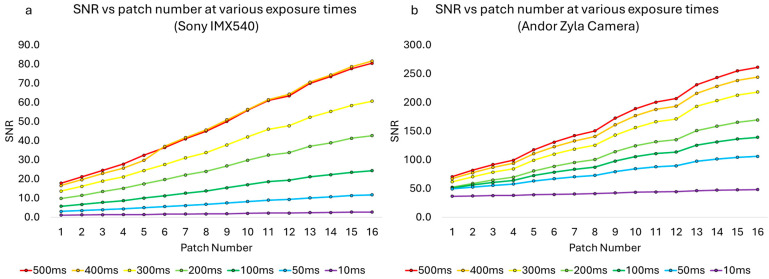
Signal-to-noise ratio vs. patch number for various exposure times. (**a**) Sony IMX540. (**b**) Andor Zyla camera.

We then plotted the signal-to-noise ratio (SNR) for all 16 patches at seven different exposure times ranging from 10 ms to 500 ms. We calculated SNR as the average intensity measured in each patch divided by the standard deviation of the background, obtained from an equal-sized patch in the corner of the image in which there was no fluorescence signal. One can see that the Andor Zyla camera reached a higher SNR, with a maximum of 261.3 for the brightest patch at 500 ms exposure, compared with an SNR of 80.5 for this same patch imaged with the IMX540 CMOS camera at 500 ms exposure. At 500 ms exposure, the difference in SNR was consistent across the different brightness patches, with an average ratio of 3.48 +/− 0.23, reflecting the difference in sensitivity between the two cameras (note this does not attempt to correct for the difference in pixel sizes between the cameras). This data is shown in Figure 5.

### 3.2. Resolution Measurements

We next measured the lateral and axial resolution of the system. For the lateral resolution measurements, we measured the full-width at half maximum of a Gaussian fit to images of 100 nm fluorescent nanobeads (F8800, Thermo Fisher Scientific) using maximum likelihood methods and an integrated Gaussian function [13]. For the axial resolution, we measured the full-width at half maximum of a Gaussian fit to a *Z*-axis intensity profile obtained from a stack of images of a thin fluorescent film. The results are shown in Table 4, and are fairly similar to those of Halpern et al. [29]. For these measurements we used the IMX540 camera and 532 nm laser with an ET575/50 emission filter.

### 3.3. Imaging of Biological Samples

**Figure 6:**
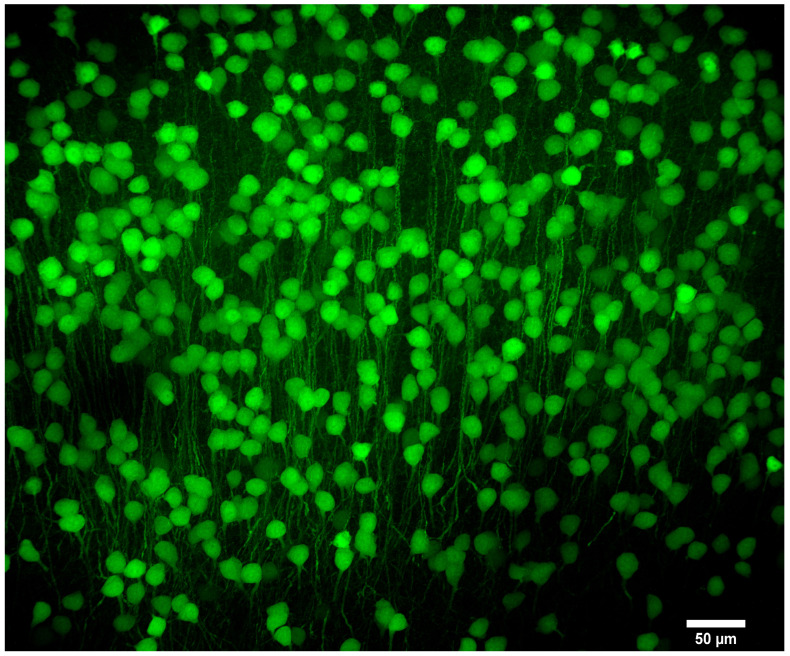
Neurons of the subiculum in the hippocampus of a *Thy1*-GFP mouse brain. Maximum intensity projection of 109 slices, Z-spacing 2 µm. Similar to Franklin and Paxinos slice 64 or Allen Mouse Brain Atlas slice 92 of 132, 20×/0.85 NA oil immersion objective, IMX540 sensor.

**Figure 7:**
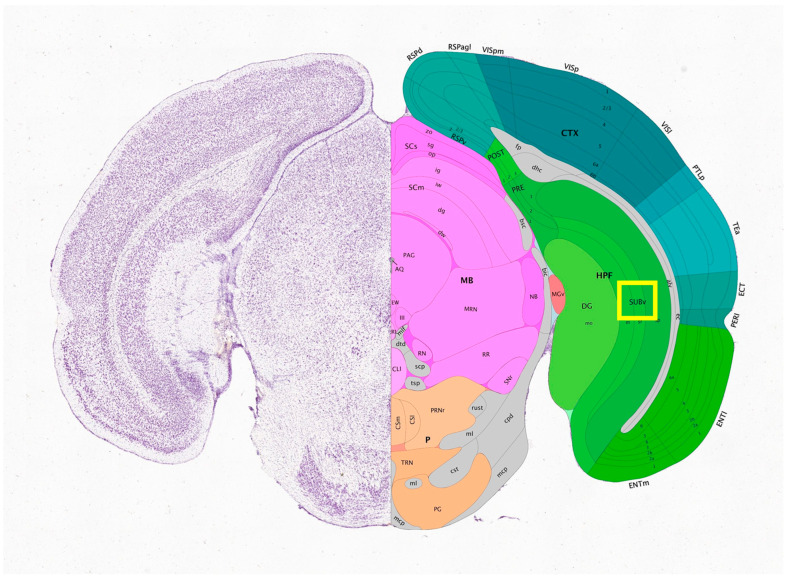
Allen Mouse Brain Atlas slice 92 of 132. Yellow square indicates the approximate imaging area.

We first imaged an optically cleared coronal mouse brain slice in which a subset of neurons express GFP [37]. This slice was matched to Paxinos and Franklin’s mouse brain atlas [38] to identify which section of the brain was being imaged. We matched our slice with slice 64 from the atlas. We further matched our sample to slice 92 of 132 in the Allen brain atlas [39,40]. Polynomial fits were made for both the horizontal and vertical directions using the slice edges and the central aqueduct as reference points. This allowed any point on this brain slice, recorded from the microscope stage coordinates, to be translated into the coordinates of the atlas for this particular slice. This method placed the neurons shown in Figure 6 in the subiculum region of the hippocampus. Figure 6 shows a maximum intensity projection of 109 optical sections acquired with a spacing of 2 µm using a 20×/0.85 oil immersion objective. This region is indicated in purple in Figure 7, taken from the Allen brain atlas (Allen Mouse Brain Atlas, mouse.brain-map.org and atlas.brain-map.org). A yellow square indicates the approximate imaging area.

**Figure 8:**
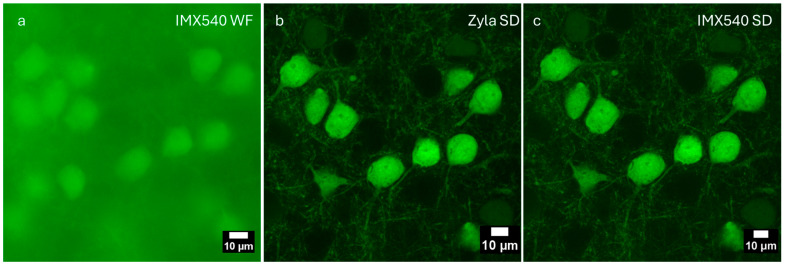
Comparison of widefield (WF) and spinning disk confocal microscopy on a single Z-slice. (**a**) Widefield fluorescence image captured with the Sony IMX540 sensor. (**b**) Spinning disk confocal image captured with the Andor Zyla camera. (**c**) Spinning disk confocal image captured with the Sony IMX 540 sensor.

We compared the Sony IMX 540 sensor with the Andor Zyla sensor on a single Z-slice of the *Thy1*-GFP mouse brain sample. This is shown in Figure 8. Figure 8a shows a widefield fluorescence image captured with the Sony IMX540 sensor, Figure 8b shows a spinning disk confocal image captured with the Andor Zyla camera, and Figure 8c shows a spinning disk image in the same region captured with the Sony IMX 540 sensor. Figure 8b,c demonstrate the optical sectioning capability of the spinning disk system. This sample is approximately 200 µm thick and is very dense with GFP-expressing neurons. Widefield microscopy is nearly useless in this case. This can be seen in Figure 8a, where all of the fine details are lost because of the overwhelming out-of-focus fluorescence signals reaching the detector. Comparing Figure 8b,c, one can see that the IMX540 sensor has equivalent image quality to the Andor Zyla camera.

**Figure 9:**
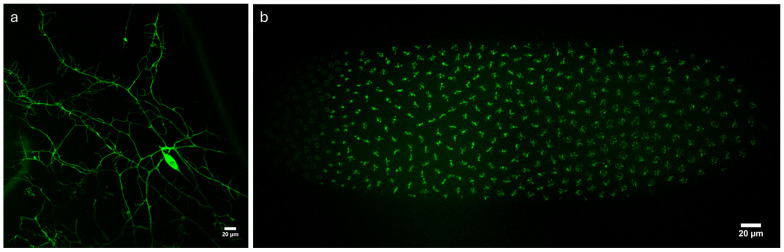
Imaging *Drosophila melanogaster*. (**a**) *Drosophila* larvae expressing GFP-labeled dendritic arborization neurons. Single optical section. (**b**) *Drosophila* embryo expressing GFP-labeled histones. Maximum intensity projection of 73 slices, spacing 0.5 µm. For both images, 20×/0.85 NA oil immersion objective, IMX540 sensor.

To further illustrate the usefulness of the system in biological research, we next imaged *Drosophila melanogaster* larvae and embryos expressing GFP-labeled structures. Figure 9a shows a single optical section of a *Drosophila* larvae expressing GFP-labeled dendritic arborization sensory neurons [32,33]. Figure 9b shows a *Drosophila* embryo expressing GFP-labeled Histone 2Av in the early stages of development, approximately 1 h after fertilization at about the 9th cell cycle [41]. The level of GFP expression in these samples is much lower in this sample compared to the *Thy1*—GFP mouse brain, demonstrating that the system is capable of imaging at lower light levels.

**Figure 10:**
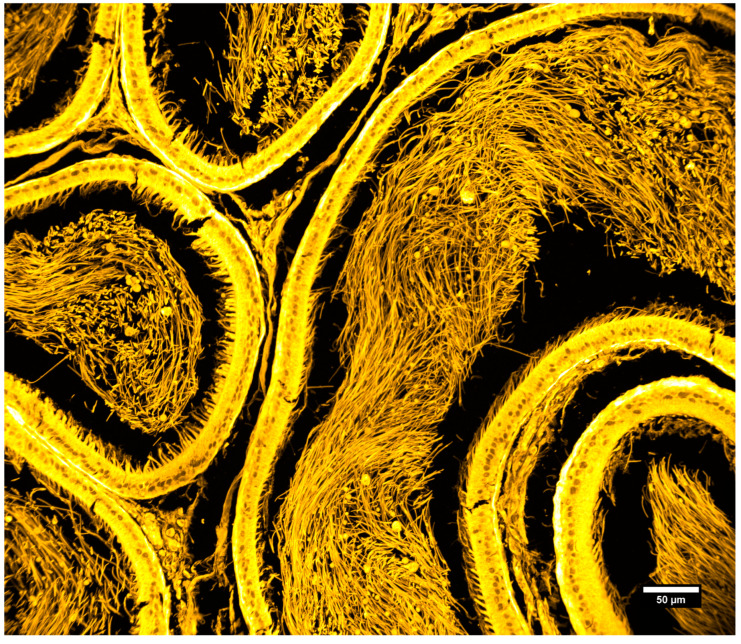
Rat testis, prepared slide. Maximum intensity projection of 69 slices, Z-spacing 0.5 um. Objective: 20×/0.85 NA oil immersion, IMX540 sensor.

**Figure 11:**
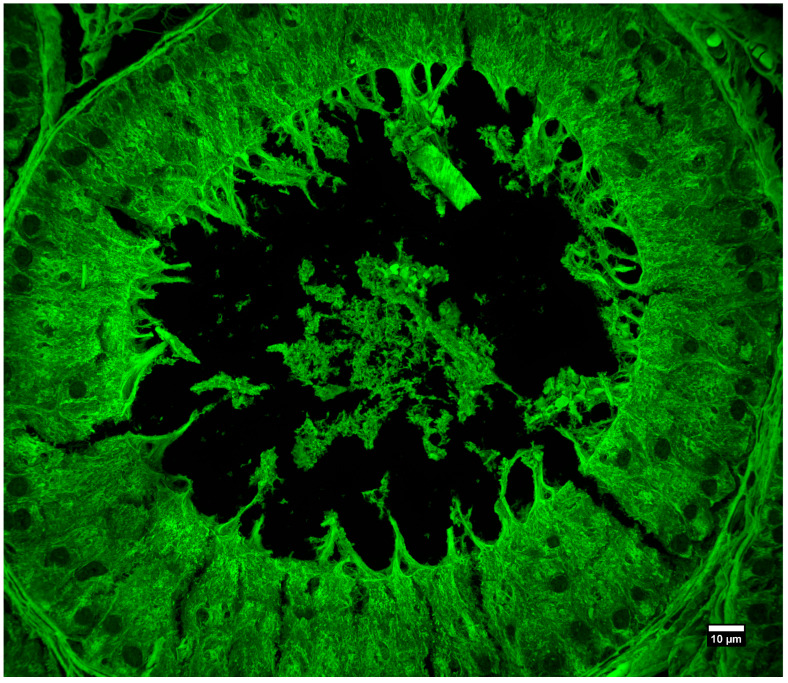
Rat testis, prepared slide. Maximum intensity projection of 72 slices, Z-spacing 150 nm. Objective: 60×/1.42 NA oil immersion, IMX540 sensor.

We also imaged commercially available prepared slides containing rat testis tissues that were stained with hematoxylin and eosin (slide number 31-6464, Carolina Biological, Burlington, NC, USA). This preparation is highly fluorescent and contains tissues with intricate 3D structures. Figure 10 and Figure 11 show two different areas of the sample with two different magnifications and illustrate the high-resolution imaging capabilities of the system over a large field of view.

**Figure 12:**
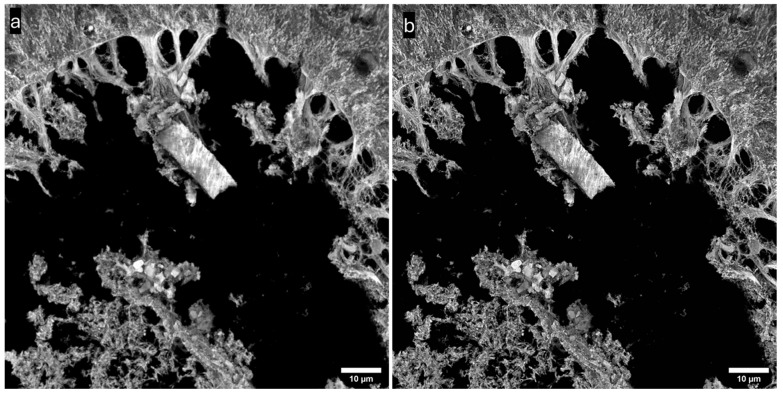
Rat testis, prepared slide. Maximum intensity projection of 72 slices, Z-spacing 150 nm. Objective: 60×/1.42 NA oil immersion, IMX540 sensor. (**a**) Spinning disk, (**b**) after deconvolution.

We finally examined the effect of deconvolution. We used Richardson–Lucy deconvolution with 10 iterations as executed in Deconvolution Lab 2 [42] running in FIJI [12]. We used a theoretical point spread function (with a Born and Wolf model) calculated by the PSF generator plugin [43] for FIJI. The results are shown in Figure 12, which shows a comparison of spinning disk confocal microscopy before (Figure 12a) and after deconvolution methods were applied (Figure 12b). Further sharpening of the image after deconvolution is apparent

## 4. Conclusions

Lower-cost designs for advanced fluorescence microscopy applications such as spinning disk confocal microscopy are expected to increase their accessibility and use by a greater number of researchers. The spinning disk module, lasers, and detectors are all costly components. Here we demonstrated a complete system in which these three components were all replaced with lower-cost alternatives, for a cost savings of about 45× less than some commercial offerings.

One example where the high cost of scientific CMOS cameras has limited their use is in multi-camera setups, which has sometimes led to complex optical arrangements in order to direct the multiple images onto one camera. Some examples where multiple images were routed onto a single camera includ certain implementations of the programmable array microscope [44,45] and the dual objective microscope for single molecule localization microscopy [34,46]. Even very recent designs have faced this issue [47].

The 25-megapixel camera used here together with the spinning disk offers large, highly detailed, optically sectioned images. Image stitching methods would usually be required to achieve the same result. Deconvolution methods were also successful, with the oversampled, high-SNR images of benefit in this approach.

## Figures and Tables

**Table 1:** Optimal pinhole size for confocal spinning disk microscopy at 515 nm.

Objective Mag.	Objective NA	Optimal Pinhole Diameter for λ = 515 nm (μm)
100	1.40	44.88
60	1.42	26.55
30	1.05	17.95
20	0.85	14.78
10	0.40	15.71

**Table 2:** Camera parameters and comparison.

Camera	FLIR Blackfly S	Andor Zyla 4.2+
Part number	BFS-U3-244S8M-C	ZYLA-4.2P-CL10
Sensor type	Sony IMX540 (Pregius S)	Andor Zyla
Quantum efficiency	69% at 525 nm	82% at 555 nm
Read noise	2.31 e^−^	0.9 e^−^
Dark current	0.3 e^−^/pixel/s	0.1 e^−^/pixel/s
Pixel size	2.74 um	6.5 um
Full well capacity	9648 e^−^	30,000 e^−^
Pixel format	5320 × 4600 (24.5 MP)	2048 × 2048 (4.2 MP)
Maximum frame rate	15 FPS	100 FPS
Readout method	Global shutter	Rolling shutter
CMOS type	Back-illuminated	Front-illuminated
Sensor size	14.599 × 12.626 mm	13.3 × 13.3 mm
Interface	USB3	Camera link
Weight of camera	53 g	1 kg
Size of camera	29 × 29 × 39 mm	133 × 80 × 82 mm
Approx. price	USD2225	USD 18,000

**Table 3:** Sampling rate consideration for various objectives considering a widefield microscope.

Objective Mag/NA	Expected Res. at 515 nm (nm)	Pixel Size (Sony, nm)	Sampling Rate (Sony)	Over- Sampled? (Sony)	Pixel Size Andor (nm)	Sampling Rate (Andor)	Over- Sampled? (Andor)
10×/0.40	785	274	2.87	Yes	650	1.21	No
20×/0.85	370	137	2.70	Yes	325	1.14	No
30×/1.05	299	91	3.28	Yes	216	1.38	No
40×/0.95	330	68.5	4.83	Yes	162.5	2.03	Yes
60×/1.35	233	45.6	5.10	Yes	108	2.14	Yes
60×/1.42	221	45.6	4.85	Yes	108	2.05	Yes
100×/1.40	224	27.4	8.19	Yes	65	3.45	Yes

**Table 4:** Resolution measurements.

Objective Mag/NA	XY	Z
20×/0.85	384.8 nm	1.932 μm
60×/1.42	215.8 nm	521.9 nm

## Data Availability

Data is available upon reasonable request.

## Abbreviations

The following abbreviations are used in this manuscript:

NA	Numerical Aperture
MP	Megapixel
CMOS	Complementary metal oxide semiconductor
GFP	Green fluorescent protein
H&E	Hematoxylin and eosin
WF	Widefield
SD	Spinning disk
DPSS	Diode-pumped solid state
WD	Working distance
